# The changes in biventricular remodelling and function after atrial septal defect device closure and its relation to age of closure

**DOI:** 10.1186/s43044-020-00120-x

**Published:** 2020-12-09

**Authors:** Areej Alkhateeb, Alaa Roushdy, Hosam Hasan-Ali, Yehia Taha Kishk, Ayman K. M. Hassan

**Affiliations:** 1 Cardiology Division of Internal Medicine Department, South Valley University Hospital, Qena, 83523 Egypt; 2 King’s College London, London, UK; 3 Congenital and Structural Heart Disease Unit, Department of Cardiology, Ain Shams University Hospitals, Cairo, Egypt; 4 Department of Cardiology, Assiut University Hospitals, Assiut, Egypt

**Keywords:** Atrial septal defect, ASD device closure, Left and right ventricular global longitudinal strain, Diastolic function

## Abstract

**Background:**

The trans-catheter closure of atrial septal defect (ASD) usually has a rapid impact on biventricular remodelling and functions. Whether the transcatheter closure of ASD at early childhood or at adulthood age would affect the improvement in biventricular dimensions and functions remains an area of active research.

**Results:**

This prospective observational study enrolled 70 subjects (50 ASD cases and 20 control subjects). Tissue Doppler imaging (TDI) and strain (S) were performed for the control group and ASD patients at baseline and at 24 h and 1 month after ASD device closure. The total ASD group was subdivided into two subgroups: group-1—children and adolescent with ASD, who underwent transcatheter closure at age ≤ 19 years; group-2—adult who underwent ASD device closure at age > 19 years old. The right and left ventricular global longitudinal systolic strain (RV/LV-GLS) and RV free wall longitudinal strain (RV free wall LS) showed a significant decline after 24 h of device closure (RVGLS-P = 0.001, LVGLS-P = 0.048, RV free wall LS-P < 0.001). However, after a 1-month follow-up, the LVGLS increased in comparison with 24 h changes after device closure (*P* = 0.038). The baseline mean value of RV free wall LS of G2 was significantly lower than G1 value (*P* < 0.001). There was no statistically significant difference between the 2 age subgroups regarding biventricular GLS and RV free wall LS changes after device closure. The changes in LV diastolic function immediately and after 1 month of device closure showed a statistically significant change in *e′* and its delta change value in group-2 in comparison with its baseline values and to group-1 delta changes (*P* = 0.002, *P* = 0.011, *P* = 0.019, respectively).

**Conclusion:**

The ASD transcatheter closure reduced biventricular global and RV free wall longitudinal systolic strain within 1 day of intervention and was associated with a short-term improvement in the LV-GLSS after a 1-month duration. The progressive increase in LV preload results in its strain growth and reduction in diastolic function after transcatheter ASD closure. The older age at the time of ASD device closure was associated with a significant decrease in the RV free wall LS and septal *e′* velocity towards abnormality.

## Background

The typical recorded effect of atrial septal defect (ASD) on ventricular remodelling and function was the RV volume overload and dysfunction under extreme conditions, which in a few cases may lead to LV dysfunction [1]. Device closure of ASD immediately increases the blood volume flow to the left ventricle and may unmask subtle abnormalities in systolic and diastolic functions. Currently, clinical research in cardiac mechanics is moving from short- and long-axis LV and RV function and ejection fraction to two- and three-dimensional (2D and 3D) ventricular deformation studies (strain and strain rate quantification) [2]. These methods allow quantification of myocardial motion and deformation in different directions (longitudinal, radial and circumferential), whilst conventional methods mainly rely on the assessment of radial function. Strain imaging has also been used to demonstrate that patients who underwent device closure of an ASD had better LV and RV longitudinal deformation than patients who underwent surgical closure of an ASD [3]. However, the optimal time of ASD closure was studied by many researchers to make a balance between the early benefit of unloading of the RV and the safety of operation [4–6]. According to the previous literature, the closure of ASD should be delayed in asymptomatic infant until the age of 2–4 years old [7]. Some investigators found that any ASD > 8 mm was considered an exception for the previous recommendation and indicated for closure at early childhood to avoid overgrowth with time [8, 9]. At the same time, there was some studies identified that the early closure of ASD by transcatheter or surgery had some complications related to access site haematoma and cardiovascular and systemic comorbidities [10]. On the other hand, closure of ASD in elderly especially after the age of 40 had a higher adverse effect than in young patients due to the long-term impact of pulmonary hypertension, arrhythmia and other complications of RV volume overload [6]. Thus, the impact of device closure on LV systolic and diastolic function according to the age of ASD patient at the time of closure is still under investigation. For these reasons, the researchers sought to measure RV and LV haemodynamic changes with 2D transthoracic echocardiography (TTE) and strain quantification in ASDs before and after transcatheter closure with particular emphasis on the assessment of biventricular function and dimensional changes between the different age groups.

## Methods

### Study population and design

After approval from the institutional ethical committee and written consent of the patient and their parents, 50 ostium secundum ASD patients and 20 age-matched control healthy subjects were recruited.

The ASD patients with haemodynamically significant left to right shunt (Qp/Qs > 1.5), dilated right-sided chambers denoting volume overload and/or pulmonary hypertension, who were referred for elective transcatheter closure, were included in this prospective observational study. The inclusion criteria included the presence of isolated ostium secundum ASD with a diameter of > 5 and < 40 mm and sufficient edges (> 5 mm) except for aortic one and pulmonary vascular resistance (PVR) < 5 Wood units/m^2^ and < 2/3 systemic vascular resistance (SVR) [11].

The criteria of exclusion were those with insufficient ASD rims (except aortic rim), other types of ASD, irreversible pulmonary hypertension or PVR > 2/3 SVR and any associated condition that may result in systolic or diastolic dysfunction, such as any type of arrhythmia especially atrial fibrillation, hypertension, diabetes, ischaemic heart disease and heart failure or LV diastolic dysfunction.

For all cases, a comparison was made between the findings of the 2D-TTE-derived tissue Doppler (TDI) and strain (S) imaging before, at 24 h and 1 month after intervention.

According to the WHO and USA, age limits of childhood, adolescence and adulthood and the age of ASD patients at the time of transcatheter closure, the researchers divided the study group into two subgroups: group-1 was a group of children and adolescents whose transcatheter closure was done at age less than or equal 19 years old (*n* = 34), and group-2 included adults who underwent transcatheter closure at age > 19 years old (*n* = 16) [12–14].

### Transthoracic echocardiography

An iE33 ultrasound system (Philips Healthcare, Best, The Netherlands) equipped with a 2D cardiac probe S5–1 (1–5 MHz) was used to conduct all standard TTE imaging with ECG gated views of grey scale, M-mode, tissue Doppler (TDI), 2D colour Doppler and S and SR.

The echocardiographic views included long and short parasternal views and apical two-, three- and four-chamber views. Throughout the subcostal view, we primarily used 2D images and coloured flow images to determine the size of ASD, the anatomical characteristics of the defect and its relationship to the superior and inferior vena cava. The sample volume was set at 5 mm for continuous wave (CW) and pulsed wave (PW) Doppler images. Each view was stored in cine loop with three cycles and a frame rate of 40–80 Hz for offline analysis by using the Q-lab software following the European Society of Cardiology (ESC) speckle tracking-recommended protocols [15].

Left atrial diameter (LAD) and RV and LV end-diastolic diameters (RVEDD and LVEDD) were quantified from apical four-chamber (A4C) view and were estimated using M-mode image in the long-axis parasternal (LAP) view. Consequently, the RVEDD/LVEDD ratio was obtained. Tricuspid annular plane systolic excursion (TAPSE) has been acquired from the lateral point of the tricuspid annulus in A4C view through the M-mode approach [16]. In parasternal long-axis view, the left ventricular and aortic dimensions were measured on a parasternal long-axis view. Additionally, 2D-TTE and derived Doppler measurements were used to estimate the pulmonary/systemic shunt ratio (Qp/Qs) through the following equation: [RVOT (RV outflow tract) VTI (velocity time integral) × RVOTd (RVOT diameter)]**/**[LVOT (LV outflow tract) VTI × LVOTd (LVOT diameter)] [17, 18]. CW Doppler echocardiography of the tricuspid flow provides the estimated systolic pulmonary artery pressures (sPAP) [19, 20]. Using tricuspid regurgitation jet velocity (*V*) and simplified Bernoulli equation, the sPAP is best derived from RV systolic pressure (RVSP): RVSP = 4(*V*)2 + derived RA pressure [21].

#### LV diastolic function assessment

Tissue Doppler imaging (TDI) was estimated at the basal-lateral and septal mitral annulus from the A4C view to calculate the early diastolic velocity (*e′*) and late diastolic velocity (*a′*) (normal values: septal *e′* = 8 mm/s, lateral *e′* = 10 mm/s, respectively) [22].

#### 2D strain imaging

The examination of patients and healthy subjects was done in the left lateral position before, at 24 h and 1 month after the ASD closure. Then, the 2D harmonic images of LV and RV for later offline processing were recorded. For offline analysis, 2D strain data were collected and processed in a cine loop format (movie clips) in all apical views. In the apical two-, three- and four-chamber views, the left ventricle’s endocardium was drawn automatically in the end-systole over the entire heart cycle. Afterwards, the RV endocardial borders were tracked manually then divided automatically into seven segments by the same software of LV (basal, mid, apex and apical segments of the septum and lateral wall), where the septum was shared between both ventricles and the average RV free wall longitudinal strain was measured as a mean value of three segments of apical, mid- and basal lateral wall.

The Q-lab data analysis (Philips) software showed the global and regional peak longitudinal S for the respective segments of the LV and RV (GLS, PLSS, respectively) after approved of the cine loops by the read operator.

### Perioperative assessment

In the perioperative assessment of ASD patients, TEE was performed for all patients to assess the suitable device size. All patients aged more than 40 years underwent the diagnostic coronary angiography before intervention.

### Procedural details

Under general anaesthesia, the transcatheter closure of the ASD was carried out with fluoroscopic and TEE assessment. In patients with ASD and pulmonary hypertension, the assessment of pulmonary artery pressure and PVR was done under 100% FiO_2_ before ASD closure. According to the exclusion criteria, all patients with elevated left atrial pressure (LAP) and left ventricular end-diastolic pressure (LVEDP) > 12 mmHg at baseline were assumed to have LV diastolic dysfunction and were excluded from the study [23]. In the present study, device size was chosen after adding 2–4 mm to the widest defect diameter according to TEE guidance or by using the balloon stretching diameter of transcatheter stop-flow technique. Then, the selected Amplatzer Septal Occluder (AGA Medical, Golden Valley, MN, USA) was implanted. For complex cases, the balloon-assisted technique of device deployment was used.

### Statistical analysis

The researchers used the Statistical Package for the Social Science (SPSS) 24.0 (IBM) for statistical analysis. The histogram and Q-Q plot were used for testing the normality of data. Continuous variables were presented as mean ± standard error. Comparison between the different groups was done by using independent two-tailed *t* test. The paired *t* test was used to compare the results of the same group before and after the intervention. The statistically significant data was considered when *P* value < 0.05.

## Results

### Patients characteristics

All the ASD and age-matched control subjects’ demographic data were presented in Table 1. The ASD cohort had a mean defect size of 1.4 ± 0.09 cm and a mean device size of 1.9 ± 0.1 cm (Table 1).

**Table 1:** Baseline demographic data of the ASD and control cases

	ASD (***N*** = 50)	Control (***N*** = 20)
**Age (years)**	16.4 ± 2.4	18.2 ± 2
**Female gender (*n*** **(%))**	28 (56%)	17 (85%)
**Height (m)**	1.24 ± 0.05	1.5 ± 0.05
**Weight (kg)**	39.4 ± 3.9	55.6 ± 4.4
**BSA (m**^**2**^**)**	1.06 ± 0.08	1.4 ± 0.08
**HR (bpm)**	96.6 ± 2.7	85.3 ± 2.1
**Mean defect size (cm)**	1.4 ± 0.09	_
**Mean device size (cm)**	1.9 ± 0.1	_

Continuous data was expressed as mean ± SEM; categorical data was presented as numbers and percentage

*BSA* means body surface area, *HR* heart rate, *N* number

### Baseline and early standard echocardiographic changes post-ASD device closure

Transcatheter closure of ASDs was successfully performed in all patients using the Amplatzer Septal Occluder devices without any significant complications. None of the trivial residual shunts at the end of the procedure was detected at 24 h after closure. There was a significant increase in RVEDD, RV/LV ratio, TAPSE, RVSP and Qp/Qs in ASD cases more than the control subjects’ values; however, they had a lower LAD and LVEDD than the control subjects (Table 2).

**Table 2:** Baseline standard transthoracic echocardiographic measures of ASD versus control groups

Basic parameters	ASD	Control	***P*** value
**LAD (cm)**	2.8 ± 0.1	3.1 ± 0.1	0.6
**LVEDD (cm)**	3.5 ± 0.1	4.6 ± 0.1	**< 0.001**
**RVEDD (cm)**	3.07 ± 0.1	1.7 ± 0.05	**< 0.001**
**RVEDD/LVEDD (cm)**	0.8 ± 0.03	0.38 ± 0.01	**< 0.001**
**Septal** ***e′*** **(cm/s)**	11.4 ± 0.3	11.5 ± 0.3	0.610
**TAPSE (cm)**	2.7 ± 0.08	2.3 ± 0.1	**0.003**
**RVSP (mmHg)**	30.3 ± 1.3	21.3 ± 1.0	**< 0.001**
**Qp/Qs**	2.83 ± 0.16	0.84 ± 0.03	**< 0.001**

Reported data were presented as mean values ± SEM. *P* value < 0.05 was considered significant. *P* value < 0.001 was considered highly significant. Independent two-tailed *t* test was used for comparison between the groups

*LAD* left atrial diameter, *LVEDD* left ventricular end-diastolic diameter, *RVEDD* right ventricular end-diastolic diameter, *Septal e′* early diastolic velocity of septal mitral annulus motion, *TAPSE* tricuspid annular plane systolic excursion, *RVSP* right ventricular systolic pressure, *Qp/Qs* systemic/pulmonary blood flow

Changes in LVEDD and RVEDD and, therefore, RV/LV ratio, 24 h and 1 month after ASD device closure, were significant (Table 3). TAPSE was reduced acutely 24 h after ASD device closure and then increased, but all changes were within a normal range after 1 month. Furthermore, there was a progressive decrease in RVSP and Qp/Qs.

**Table 3:** Standard transthoracic echocardiographic measurements at baseline, 24 h and 1 month post-ASD closure

Basic parameters	Pre-closure	Post-closure 1	Post-closure 2	*P*value1	*P*value2	*P*value3
**LAD (cm)**	2.85 ± 0.13	2.83 ± 0.12	3.06 ± 0.12	0.701	0.690	0.960
**LVEDD (cm)**	3.55 ± 0.13	3.71 ± 0.13	4.15 ± 0.13	**0.039**	**0.001**	**0.017**
**RVEDD (cm)**	3.07 ± 0.12	2.75 ± 0.12	2.53 ± 0.13	**< 0.001**	**< 0.001**	**< 0.001**
**RVEDD/LVEDD (cm)**	0.88 ± 0.03	0.75 ± 0.02	0.60 ± 0.02	**0.003**	**< 0.001**	**< 0.001**
**Septal** ***e′*** **(cm/s)**	11.42 ± 0.37	9.66 ± 0.34	9.15 ± 0.49	**< 0.001**	**0.002**	0.950
**TAPSE (cm)**	2.74 ± 0.08	2.48 ± 0.07	3.19 ± 0.50	**0.003**	0.420	0.210
**RVSP (mmHg)**	30.36 ± 1.38	23.94 ± 1.03	23.22 ± 1.16	**< 0.001**	**< 0.001**	0.110
**Qp/Qs**	2.83 ± 0.16	0.88 ± 0.01	0.88 ± 0.02	**< 0.001**	**< 0.001**	0.58

*P*value1: ASD data (24 h after closure) in comparison with Pre-closure

*P*value2: ASD data (1 month after closure) in comparison with Pre-closure

*P*value3: ASD data (1 month after closure) in comparison with 24 h after closure

Continues data were presented as mean ± SE. *P* < 0.05 was considered significant, and < 0.001 was considered highly significant. Paired two-tailed *t* test was used for comparison between the different stages

*Pre-closure* before ASD device closure, *Post-closure 1* 1 day after ASD device closure, *Post-closure 2* 1 month after the intervention, *LAD* left atrial diameter, *LVEDD* left ventricular end-diastolic diameter, *RVEDD* right ventricular end-diastolic diameter, *Septal e′* early diastolic velocity of septal mitral annulus motion, *TAPSE* tricuspid annular plane systolic excursion, *RVSP* right ventricular systolic pressure, *Qp/Qs* systemic/pulmonary blood flow

**Fig. 1:**
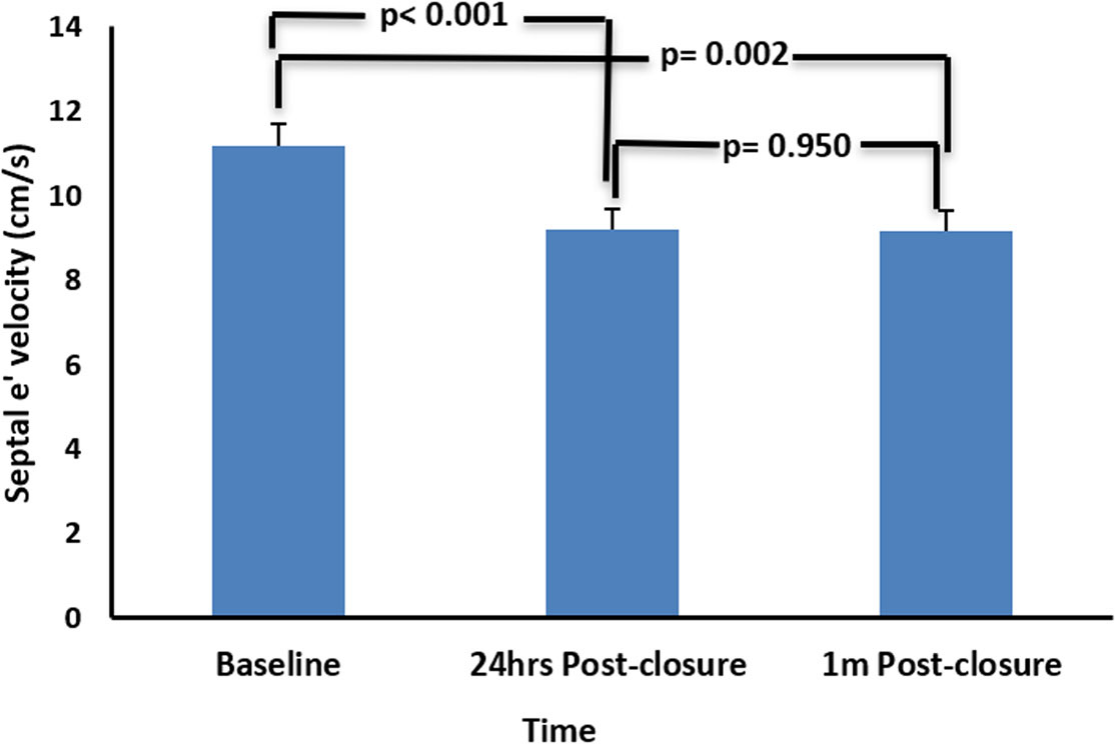
Changes in left ventricular diastolic function before and after transcatheter ASD device closure of the total ASD study group. Significant reduction in septal (*e′*) 24 h and 1 month after ASD device closure with insignificant changes between 24 h and 1 month later. ASD, atrial septal defect; LV, left ventricle; Septal *e′*, early diastolic velocity of septal mitral annulus motion

Regarding LV diastolic function, there was a significant reduction in the early diastolic velocity of septal *e′* after 24 h and 1 month in comparison with the baseline level but was within the normal range. This decrease in diastolic function was unchanged throughout the follow-up duration of 1 month (Fig. 1).

### LV- and RV-GLS and RV free wall LS changes

**Fig. 2:**
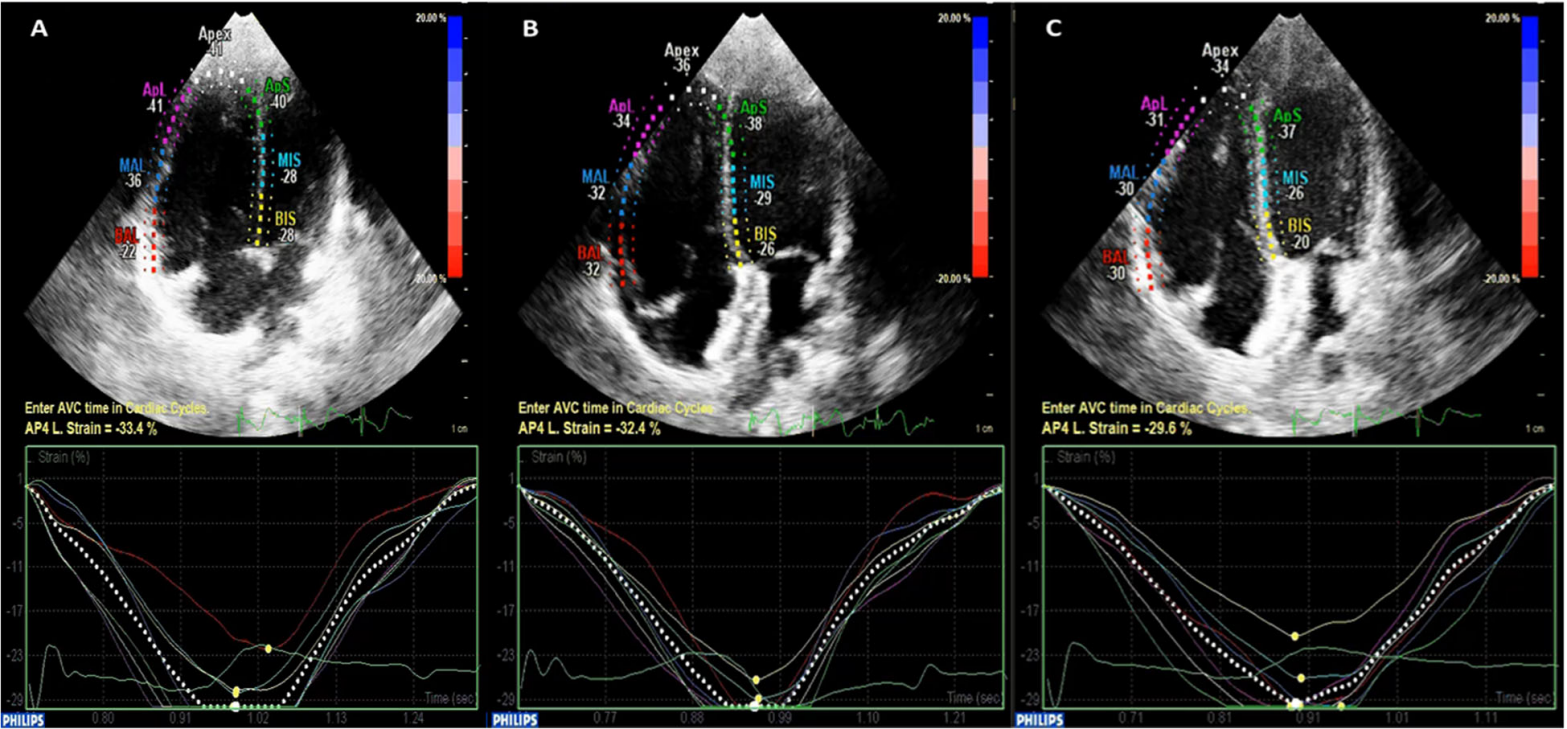
2D-TTE of the RVGLS changes in an ASD case before and after device closure. **a** Hyperkinetic RV before ASD device closure with RVGLS in apical 4 chambers view (− 33.4%). **b** Reduction in RVGLS at 24 h post-ASD device closure in the same view (− 32.4%). **c** Progressive insignificant reduction in RVGLS 1 month post-ASD device closure in the same view (− 29.6%). AP4L, apical 4 chambers longitudinal view; ApL, apical lateral; ApS, apical septum; AVC, aortic valve closure; BAL, basal anterolateral; BIS, basal inferoseptal; MAL, mid-anterolateral; MIS, mid-septum; ASD, atrial septal defect; GLS, global longitudinal strain; RV, right ventricle

There was a significant increase in the GLS of the LV in the whole study group compared to the controls and after 1 month of closure versus 24 h (Table 4). Moreover, RV/LV-GLS decreased significantly (RVGLS, from − 24.55 ± 0.79% to − 21.94 ± 0.78%, *P* = 0.001; LVGLS, from − 24.47 ± 0.57% to − 23.14 ± 0.65%, *P* = 0.048) after 24 h of ASD device closure (Fig. 2, Table 4). In the total ASD group, there was a significant reduction in average RV free wall longitudinal strain after 24 h of ASD device closure in comparison with baseline level (Δ1, − 2.79 ± 0.70%, *P* < 0.00; Table 4). Whilst the delta changes in RV free wall LS after 1 month in comparison with 24 h and baseline or all levels at the baseline, 24 h and 1 month in comparison with control levels were insignificant (Table 4).

**Table 4:**
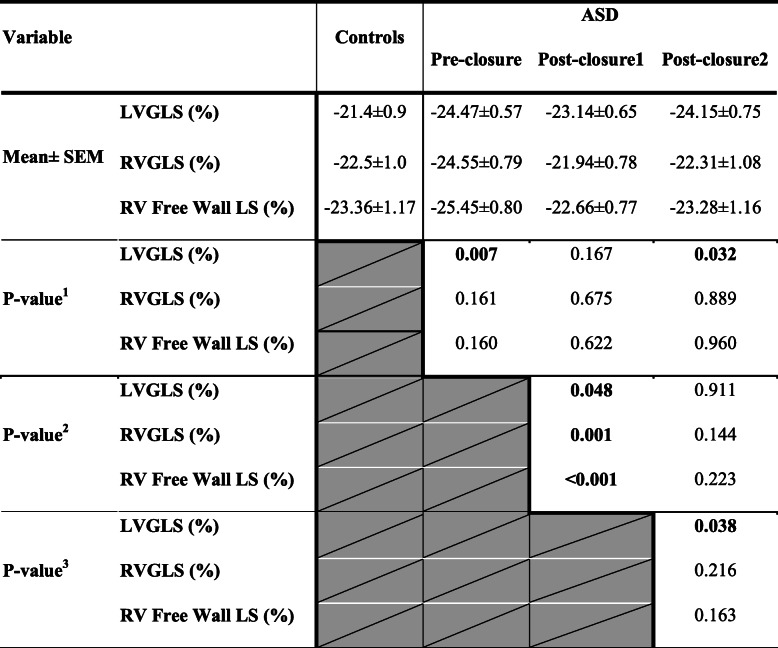
Comparison between LV and RV GLSS and RV free wall LS before, after 24 h and 1 month of transcatheter ASD closure in relation to the controls

*P*value1: Comparison of ASD data (Pre-closure, 24 h and 1 month Post-closure) versus controls

*P*value2: ASD data (24 h and 1 month Post-closure) in comparison with Pre-closure

*P*value3: ASD data (1 month after closure) in comparison with 24 h Post-closure

Continues data were presented as mean ± SEM. *P* < 0.05 is considered significant. Independent two-tailed *t* test was used to compare between ASD and controls and paired two-tailed *t* test between the 3 stages of ASD (Pre-closure versus 24 h and 1 month Post-closure)

*Pre-closure* before ASD device closure, *Post-closure1* 1 day after ASD device closure, *Post-closure2* 1 month after the intervention, *LS* longitudinal strain, *LVGLS* left ventricle global longitudinal systolic strain, *RVGLS* right ventricle global longitudinal systolic strain

### Comparison between baseline biventricular function, indexed dimensions and post-closure changes in the two age subgroups

After the subdivision of the study group into 2 subgroups according to the age at time of intervention, subgroup-1 with a mean age of 6.39 ± 0.84 years old (maximum 19, minimum 1, median 4, *n* = 34) had a significantly larger LA, LVED and RVED diameters indexed to BSA in comparison with subgroup-2 with a mean age of 37.66 ± 3.55 years old (maximum 67, minimum 21, median 37.8, *n* = 16) at baseline, whilst subgroup-2 had a significantly higher RVSP and TAPSE (Table 5). The mean defect size and device size were significantly lower in subgroup-1 compared to subgroup-2 (1.18 ± 0.08 cm and 1.60 ± 0.10 cm, *P* < 0.001, versus 2.01 ± 0.15 cm and 2.79 ± 0.14 cm, *P* < 0.001, respectively). However, there was insignificant difference between the 2 age groups in the rate of acute changes in TAPSE 24 h and 1 month after ASD device closure (*P* value = 0.459, 0.272, respectively)

**Table 5:** Demographic data and baseline 2D-TTE parameters of the 2 ASD subgroups

Basic parameters	Group-1 (***n*** = 34)	Group-2 (***n*** = 16)	***P*** value
**Age (years)**	6.39 ± 0.84	37.66 ± 3.55	**< 0.001**
**Female gender (*n*** **(%))**	16 (47.1%)	12 (75%)	0.065
**Height (m)**	1.05 ± 0.04	1.63 ± 0.03	**< 0.001**
**Weight (kg)**	23.48 ± 2.74	73.31 ± 3.69	**< 0.001**
**BSA (m**^**2**^**)**	0.79 ± 0.06	1.78 ± 0.05	**< 0.001**
**HR (bpm)**	104 ± 2.96	81.06 ± 3.23	**< 0.001**
**Mean defect size (cm)**	1.18 ± 0.08	2.01 ± 0.15	**< 0.001**
**Mean device size (cm)**	1.60 ± 0.10	2.79 ± 0.14	**< 0.001**
**LADi (cm/m**^**2**^**)**	3.22 ± 0.12	2.18 ± 0.08	**< 0.001**
**LVEDDi (cm/m**^**2**^**)**	4.29 ± 0.16	2.45 ± 0.08	**< 0.001**
**RVEDDi (cm/m**^**2**^**)**	3.68 ± 0.19	2.24 ± 0.08	**< 0.001**
**RVEDDi/LVEDDi (cm/m**^**2**^**)**	0.87 ± 0.04	0.92 ± 0.03	0.435
**TAPSE (cm)**	2.61 ± 0.09	3.00 ± 0.16	**0.034**
**RVSP (mmHg)**	27.38 ± 1.31	36.68 ± 2.75	**0.001**
**Qp/Qs**	2.74 ± 0.22	3.01 ± 0.22	0.462
**Septal** ***e′*** **(cm/s)**	11.71 ± 0.36	10.82 ± 0.87	0.270
**LVGLS (%)**	− 25.87 ± 0.67	− 21.50 ± 0.58	**< 0.001**
**RVGLS (%)**	− 26.51 ± 0.84	− 20.38 ± 1.21	**< 0.001**
**RV free wall LS (%)**	− 27.45 ± 0.83	− 21.20 ± 1.23	**< 0.001**

Reported data were presented as mean values ± SEM. *P* value < 0.05 was considered significant. Independent *t* test was used for comparison between the groups

*Group-1* children and adolescent with ASD, *Group-2* adults with ASD, *HR* heart rate, *BSA* body surface area, *LADi* indexed left atrial diameter/BSA, *LVEDDi* indexed left ventricular end-diastolic diameter/BSA, *RVEDDi* indexed right ventricular end-diastolic diameter/BSA, *Septal e′* early diastolic velocity of septal mitral annulus motion, *TAPSE* tricuspid annular plane systolic excursion, *RVSP* right ventricular systolic pressure, *Qp/Qs* systemic/pulmonary blood flow, *LS* longitudinal strain, *LVGLS* left ventricle global longitudinal strain, *RVGLS* right ventricle longitudinal strain

**Fig. 3:**
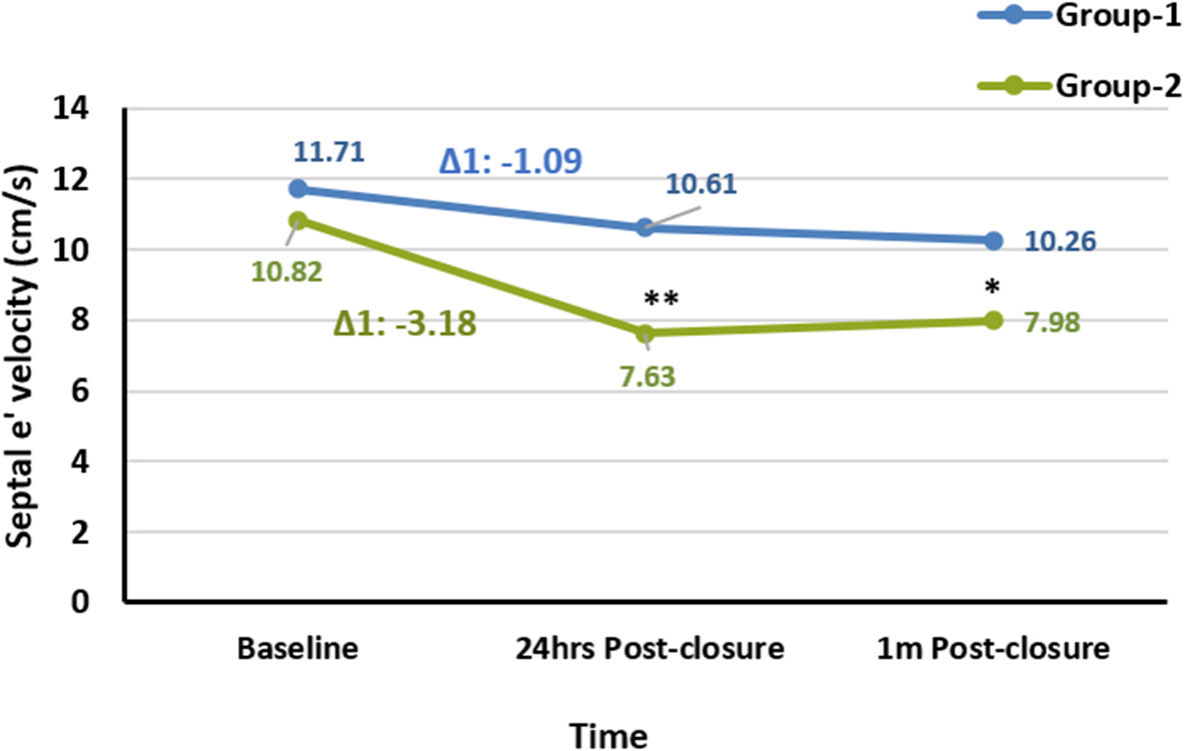
Comparison of the changes in left ventricular diastolic function (septal *e′* velocity) after ASD device closure between the 2 age groups and along the time course before and after closure. A linear chart of septal *e′* velocity changes from baseline towards 24 h and 1 month post-closure in children and adolescent group-1 and marked reduction with abnormal values (7.63, 7.98 cm/s) in adult group-2 (G1-Δ1 − 1.09 cm/s versus G2-Δ1 − 3.18 cm/s, *P* = 0.019). Δ1 = difference between 24 h post-ASD device closure and baseline values. **A highly significant difference between the 1st and 2nd groups after 24 h of ASD device closure in septal *e′* velocity (*P* < 0.001). *A significant difference between the 2 groups after 1 month of ASD device closure (*P* = 0.001). Group-1: children and adolescent with ASD who underwent closure at age ≤ 19 years old; group-2: adult with ASD who underwent closure at age > 19 years old. ASD, atrial septal defect; septal *e′*, early diastolic velocity of septal mitral annulus motion

Although there was an insignificant difference between the 2 subgroups at baseline regarding the septal *e′* velocity (11.71 ± 0.36 cm/s versus 10.82 ± 0.87 cm/s, *P* = 0.270) (Table 5, Fig. 3), this difference became significant after 24 h and 1 month post-ASD device closure (*P* < 0.001, *P* = 0.001, respectively) as septal *e′* velocity showed a significant decline in subgroup 2 after 24 h, and this decline was persistent 1 month post-device closure (10.82 ± 0.87 cm/s versus 7.63 ± 0.63 cm/s, and 7.98 ± 0.72 cm/s, *P* = 0.002, 0.011, respectively) (Fig. 3). In subgroup 1, there was a significant reduction in septal *e′* velocity after 24 h and 1 month post-closure compared to baseline but was still within the normal range (11.71 ± 0.36 cm/s versus 10.61 ± 0.30 cm/s, and 10.26 ± 0.29 cm/s, *P* = 0.015, 0.002, respectively) and was not as prominent as the decline in subgroup 2 (Fig. 3). The degree of reduction after 24 h in LV diastolic function in the adult’s subgroup (G-2) was significantly higher in comparison with the children subgroup (G-1) (G1-Δ1 − 1.09 cm/s versus G2-Δ1 − 3.18 cm/s, *P* = 0.019) (Fig. 3).

There was a significant difference between the LV/RV GLS in the 2 subgroups at baseline with a non-significant difference between them after ASD device closure as regards the percent change (LVGLS: Δ1-p = 0.770, Δ2-p = 0.605; RVGLS: Δ1-p = 0.824, Δ2-p = 0.735), where ∆1 means the difference change of the RV/LV global strain values at 24 h post-closure and baseline and ∆2 means the difference change between 1 month post-closure and baseline.

With regard to the mean RV free wall LS at the baseline, the adult group (G2) had a highly significant lower level than the children and adolescent group (G1) (*P* < 0.001, Table 5) with a significant reduction after 24 h in both groups (RV free wall LS: G1-Δ1 − 2.93 ± 0.92%, *P* = 0.003; G2-Δ1 − 2.50 ± 1.01%, *P* = 0.026). However, the delta changes in RV free wall LS after 1 month in comparison with 24 h and in comparison with baseline were insignificant in both age groups, with a non-significant difference between the 2 age group delta changes after 24 h and 1 month of ASD device closure (RV free wall LS: Δ1-p = 0.778, Δ2-p = 0.917).

## Discussion

In comparison with previous studies, our echocardiographic parameters demonstrated larger RV and smaller LV diameters in ASD patients, and there was a significant reduction in the RVEDD/LVEDD ratio along with a decrease in RVSP post-device closure. This was in agreement with Balci et al. who reported that closure of ASD using the Amplatzer Septal Occluder led to a decrease in RV size and an increase in LV size [24]. All these changes are mostly due to the abolishing left-to-right shunting thus reducing RV volume and increasing LV volumes post-device closure.

Regarding the impact of age at the time of closure on the RV/LV indexed dimensions and function, we identified that the LV remodelling changes because of haemodynamically significant shunt, and long-standing RV volume overload was progressive with age. Nevertheless, these changes tend to improve post-device closure irrespective of the age. On the other hand, there are some confounding factors that had impact on some baseline echocardiographic parameters, such as the positive impact of BSA and age on TAPSE to result in progressive increase in TAPSE values with adulthood and increased BSA [25]. However, the inverse relation between the BSA of underweighted ASD children and adolescents was responsible for the exaggerated impact of ASD on increasing the indexed right-sided dimensions to BSA before the time of closure [26]. Moreover, the young age and small BSA had a positive impact on GLS and RV free wall LS beside the impact of ASD, which was significantly higher in children and adolescents [27]. Generally, the BSA has a significant impact on echocardiographic parameters of growing ages than adults [26, 28].

In agreement with the preceding studies, a significant reduction in RV GLS and RV free wall LS were observed after 24 h, and this was persistent 1 month after closure [29]. This reduction is considered normalisation of the RV function because the hyperkinetic RV wall secondary to volume overload improved to become normal. The significant increase in LV-GLS in ASD cases compared with the control group was mainly due to the dimensional and geometrical changes in LV cavity and wall before closure [30]. Moreover, the LV-GLS showed a statistically significant reduction 24 h after ASD device closure, then it was followed with a significant increase after 1 month in comparison with the values of 24 h after the intervention. This is mainly due to an increase in the blood volume to the LV after ASD closure. Such a result usually happens due to the acute unmasking of LV systolic dysfunction after closure, as described by Bussadori et al. [31], and in the current study, these changes occurred irrespective of the age at the time of device closure.

Lange et al. identified that percutaneous ASD occlusion by an Amplatzer Septal device reduced longitudinal septal annular motion [32]. Most studies that focused on LV compliance and stiffness in ASD patients revealed that the elastic stiffness-stress relationship is significantly higher in patients with ASD than in normal subjects, suggesting increased chamber stiffness [33, 34]. In a previous study involving 43 ASD patients of different ages, there was a reduction in volume with an increase in LV pressure after closure of ASD, which resulted in LV diastolic dysfunction in as many as 19% of patients and, in turn, impairment of the transmitral E/A ratio and reduction in septal *e′* [35].

In the current study, the baseline diastolic function in the form of septal *e′* was not significantly different from the control group and was reduced significantly after 24 h and 1 month post-device closure. This may be explained by the effect of device closure on the shortening of the basal and mid-segments of the septal wall. Also, it could be due to the left atrial disc occupies more space in the LA according to the study of Lange et al. [32]. Furthermore, the increase in LA pressure after abolition of the shunt will affect the LV early diastolic filling capacity and compliance. This effect was more pronounced in subgroup 2 with more significant and persistent reduction in septal *e′* compared to subgroup 1 which could be explained by the long-standing haemodynamic effect of the left to right shunt and the rebound effect of device closure on the left atrial pressure which was confirmed by previous studies that showed that the LV compression by dilated RV resulted in LV elasticity reduction, which was unmasked after increase in LV preload after ASD device closure [32].

In agreement with our results, Lange et al. found that the transcatheter closure of ASD in patients with a mean age of 23 ± 20 years reduced the longitudinal septal annular motion immediately [32]. On the other hand, Makino and his colleagues in 2020 found that the reduction in diastolic function post-ASD device closure was insignificant in young adults (mean age 49 ± 19 years old) in comparison with the elder group (62.7 ± 8.2 years old) after 6 months of follow-up [36], whilst Giardini and his colleagues compared the diastolic function of 15 children aged from 2.4 to 13.8 years before and after closure of ASD and discovered the significant improvement in LV diastolic function [37]. And in all previous studies, they identified that the LV had a rapid adaptation with changing in volume during the time course of ASD with a progressive subtle changes in diastolic function and normal systolic one [38]. Therefore, during the acute stage of device closure, the improvement in LV volume was associated with the unmasking of LV diastolic dysfunction and no changes in biventricular systolic function [38].

## Conclusion

The age at the time of ASD closure had a significant impact on LV diastolic function in the form of septal *e′* velocity but did not affect the biventricular GLS. The closure of haemodynamically significant ASD is recommended regardless of the age of closure to improve the biventricular remodelling and subsequent changes in biventricular GLS, RV free wall longitudinal strain and LV diastolic function. At the same time, the early closure had better results regarding LV geometry and diastolic function. The immediate and short-term impact of ASD device closure in adults (> 19 years old) could be associated with a tendency towards decline in diastolic function. The clinical implication of these findings could affect our routine assessment of ASD patients to include thorough assessment of LV diastolic function before device closure especially in adults.

## Limitations and recommendations

Longer-term follow-up is recommended to validate the results of this study on the impact of ASD device closure in the different age groups and to determine whether the negative effect of device closure on diastolic function in adults is temporarily and will normalise later on or will be persistent.

Multi-centre study involving larger number of patients is now considered. This kind of study will enable us to divide the patients into different tiers according to age to accurately identify the ideal age for ASD closure that can achieve the best results concerning both systolic and diastolic function post-closure.

## Data Availability

The datasets used and/or analysed during the current study are available from the corresponding author on reasonable request.

## Abbreviations

ASD: Atrial septal defect
ASO: Amplatzer Septal Occluder
*e′*: Early diastolic velocity
EDD: End-diastolic diameter
GLS: Global longitudinal strain
LA: Left atrium
LS: Longitudinal strain
LV: Left ventricle
PA: Pulmonary artery
Qp/Qs: Pulmonary flow-to-systemic flow shunt ratio
RV: Right ventricle
RVSP: Right ventricular systolic pressure
S: Strain
SR: Strain rate
SPAP: Systolic pulmonary artery pressure
TAPSE: Tricuspid annular plane systolic excursion
TDI: Tissue Doppler imaging
TEE: Transoesophageal echocardiography
TTE: Transthoracic echocardiography
2D: Two-dimensional
3D: Three-dimensional
